# Main Challenges and Actions Needed to Improve Conservation and Sustainable Use of Our Crop Wild Relatives

**DOI:** 10.3390/plants9080968

**Published:** 2020-07-30

**Authors:** Johannes M. M. Engels, Imke Thormann

**Affiliations:** 1 Alliance of Bioversity International and CIAT, Maccarese, 00054 Rome, Italy; 2 Federal Office for Agriculture and Food, 53179 Bonn, Germany; imke.thormann@gmail.com

**Keywords:** crop wild relatives, biological features, conservation, use, local, national and global efforts, policy, genetic diversity, gene donors, pre-breeding, breeding, cross-sectoral collaboration

## Abstract

Crop wild relatives (CWR, plural CWRs) are those wild species that are regarded as the ancestors of our cultivated crops. It was only at the end of the last century that they were accorded a high priority for their conservation and, thus, for many genebanks, they are a new and somewhat unknown set of plant genetic resources for food and agriculture. After defining and characterizing CWR and their general threat status, providing an assessment of biological peculiarities of CWR with respect to conservation management, illustrating the need for prioritization and addressing the importance of data and information, we made a detailed assessment of specific aspects of CWRs of direct relevance for their conservation and use. This assessment was complemented by an overview of the current status of CWRs conservation and use, including facts and figures on the in situ conservation, on the ex situ conservation in genebanks and botanic gardens, as well as of the advantages of a combination of in situ and ex situ conservation, the so-called complementary conservation approach. In addition, a brief assessment of the situation with respect to the use of CWRs was made. From these assessments we derived the needs for action in order to achieve a more effective and efficient conservation and use, specifically with respect to the documentation of CWRs, their in situ and ex situ, as well as their complementarity conservation, and how synergies between these components can be obtained. The review was concluded with suggestions on how use can be strengthened, as well as the conservation system at large at the local, national, and regional/international level. Finally, based on the foregoing assessments, a number of recommendations were elaborated on how CWRs can be better conserved and used in order to exploit their potential benefits more effectively.

## 1. Introduction

Today’s cultivated crop plants have undergone more or less drastic changes since their first cultivation and domestication. The first signs of domesticating wild plant (and animal) species date back 10,500 years in Western Asia and domestication has since then been practiced in different parts of the world by different groups of people on new species [1]. The duration and intensity of this domestication process have been very variable from one crop to the other [2]. The one thing that all crops have in common is that they originated from (one or more) wild and naturally occurring species. For a number of crops, the domestication process is well known, based on archaeological finds and (experimental) research. In general, this process started with gathering in particular wild grasses and leguminous species, followed by their cultivation closer to the homestead and gradually undergoing transformation from wild into domesticated taxa [3,4,5]. In some instances, crops are the result of natural or man-made hybrids between two wild ancestor species (e.g., banana: *Musa acuminata* and *M. balbisiana*); in other cases, the wild relative is a subspecies of the cultivated crop (e.g., *Vitis vinifera*) or there is no difference between the wild and the domesticated species (e.g., the olive tree, *Olea europea* which has wild, weedy, and cultivated forms, and many forage crops), which are just two different forms of the same species. For other crops, the domestication process is much less known or even completely obscure, including which wild species might have been involved as ancestor(s) of the crop in question (e.g., *Triticum spelta*, spelt). For some crops, the domestication process is still ongoing, especially in local fruit trees [6]. Possibly the most important consequence of the domestication process is that the genetic diversity available in the crop genepool (in the narrow sense) is usually much smaller than that in the related wild species [7,8]. In this paper, we focus on the wild species that are related to our crops, i.e., the crop wild relatives (CWRs). They have in different ways contributed (genetically) to the domestication process and thus can be regarded as the ancestral species or progenitors of our present crops, and they are a valuable resource of genetic diversity and traits for plant breeding.

It has taken several years after the global initiation of systematic collecting and conserving threatened landraces of our crops, somewhere in the 1960/70’s, until CWRs were systematically included, both at the national and international level. In 1975, a global collecting program of threatened landraces and CWRs was initiated under the coordination of the International Board for Plant Genetic Resources (IBPGR) and approximately 220,000 samples were collected during more than 1000 collecting missions in more than 130 countries, largely before 1995. The collected materials were sent to and subsequently stored in selected national and regional/international genebanks around the world [9,10]. The inclusion of CWRs in collecting efforts was triggered by the observed genetic erosion, as well as by the apparent need to include more genetic diversity for the advancement of breeding programs of major crops (e.g., potato), triggered by the success of using CWRs in breeding programs, such as the tomato, for specific traits [11]. Due to breeding programs in need for more diversity, the first ‘push’ for CWR conservation came from the international CGIAR research centers, as well as some (international) breeding companies in the 1970/80’s [12].

Only during the past few decades, significant successes of transferring traits from CWRs into cultivated crops have been reported, mostly to overcome biotic stresses, such as pests and diseases, as well as abiotic stresses, such as drought tolerance [8,13]. More recently, adaptability to changing environmental conditions, in particular those caused by climate change, has also become important. Only gradually, CWRs became a priority for the more advanced national plant genetic resources centers for food and agriculture (PGRFA), such as in the USA, UK, Germany, The Netherlands, and Australia. Possibly the biggest ‘push’ for the conservation of CWRs was the advancement of molecular biology and genetic tools and techniques that greatly facilitate the transfer of traits, genes, and alleles from one species to another, almost independent of how closely they are related to each other.

The above-mentioned developments certainly had an important impact on the increasing (political) conservation priorities accorded to CWRs since the late 1980’s/early 1990’s. This has been reflected by the inclusion of CWRs in the text of the Convention on Biological Diversity (CBD) [14] and, in 2010, in the AICHI Biodiversity Targets, in particular Target 13, as well as in target 9, of its Global Strategy for Plant Conservation, where CWRs and wild food plants were accorded a high priority for conservation [15]. In almost half of the 18 priority activities of the Second Global Plan of Action (GPA II), adopted in 2011 by the Food and Agricultural Organization of the United Nations (FAO) Member Countries, it makes (again, like in the first GPA agreed upon in 1996) a special reference to CWRs and wild food plants, highlighting the need to strengthen their conservation and sustainable use [16]. More recently, CWRs have been included in the United Nations’ Sustainable Development Goals (SDG) [17]. The recent Global Assessment Report on Biodiversity and Ecosystem Services, published in 2019 by the United Nations’ Intergovernmental Science-Policy Platform on Biodiversity and Ecosystem Services (IPBES) [18], mentions CWRs explicitly as species that are important for long-term food security, helping render ecosystems more resilient to stressors including climate change, pests and pathogens, and that lack effective protection. The report highlights the decreasing number of CWRs and mentions that many hotspots of agrobiodiversity and CWRs are under threat or not formally protected.

In response to this increasing visibility and importance of CWRs in global and international political agendas since the early 1990’s, numerous projects, tools, and guidelines have been initiated and developed at local/national, regional, and global levels. Examples for the latter are the voluntary guidelines for the conservation of CWRs and wild food plants at the national level [19] or the interactive toolkit for CWR conservation planning [20].

Besides the more political framework facilitating conservation, technical and managerial considerations are also important in order to effectively include CWR species in routine conservation programs. As treated in the following sections, a number of specific requirements can be identified that determine the ability of genebanks, in particular, to cope more effectively with CWR conservation. Especially, the availability of adequate knowledge and experience to manage this very variable and sometimes extremely difficult category of genetic resources is one of the main hurdles to overcome.

It has been a long and is yet a continuous struggle to get CWRs as a high priority on, in particular, local and national conservation agendas [21,22]. Reasons for this are limited financial resources available to many conservation and use programs; the lack of technological resources to effectively exploit these resources; an increasing debate on access to and availability of PGRFA; the sometimes severe technical challenges, which the conservation of CWRs’ can cause to genebanks, due to biological peculiarities of CWRs; as well as the relatively low priorities these resources have for local people. Against this backdrop, the paper investigates the reasons for these constraints, focusing on difficulties, opportunities and synergies that characterize the conservation and use of CWRs. Furthermore, due to the biological peculiarities of CWRs, there is a need for a strong collaboration between actors operating at different levels, especially between local/national and international, as well as between different sectors, such as agriculture and environment.

## 2. Definition and Classification of CWRs

A ‘simple’ and broad definition of a CWR is that all wild species belonging to the same genus (and that coincides in most cases with the same genepool) of a given crop are treated as a crop wild relative [23]. A narrower definition refers to the genepool concept developed by Harlan and de Wet [24]. They used the easiness of crossing a given wild relative with the crop species in question as the basis for their classification. When a CWR species crosses easily with the related crop, the species is defined as a genepool I species (GP1a = cultivated form of the crop and GP1b = wild or weedy form of the crop). Wild relatives from whom genes can be transferred to the crop, but with difficulties using conventional breeding techniques, are included in genepool II. Those wild relatives that cannot be crossed with a given crop and where gene transfer is only possible using sophisticated techniques, such as embryo rescue, somatic fusion or genetic engineering, are defined as genepool III species. Although this classification is very ‘utility driven’ and from a plant breeding perspective, it makes good practical sense, as crossing barriers are a major limiting factor for the use of CWRs in conventional plant breeding.

However, for the majority of crop complexes, particularly those from tropical areas, too little information on crossability is available to use the genepool concept. Therefore, an alternative concept has been proposed by Maxted et al. [23], based on the existing taxonomic hierarchy to define to which of four recognized taxon groups a given species belongs. Taxon group TG1a corresponds to the crop, CWRs in TG1b correspond to the same species as the crop, CWRs of TG2 are in the same series or section as the crop, TG3 is the same subgenus as the crop, and CWRs of TG4 are those in the same genus. Thus, without detailed information on the reproductive isolation, this concept can be used to establish the degree of relationship between a CWR and a crop [23].

The number of CWR species account for about 21% of the world’s flora [19,25], assuming that any species belonging to the same genus as a given crop is a CWR. On that basis, it has been estimated that there are 50,000 to 60,000 CWR and wild food plant species worldwide [19]. For Europe, Kell et al. [26] found that 17,495 (8624 of them endemic), out of approximately 20,590 species, or 85% of the European flora, comprise crop and CWR species. Maxted et al. [23] argued that a more targeted list of globally important CWR species could be obtained by focusing on the crop genepool GP1b or on taxon groups TG1b and TG2, containing the closest CWR species. By applying this to genera that contain major and minor food crops, as defined by Groombridge and Jenkins [27], that the resulting 77 genera contain 10,739 CWR species that are congeneric to these genera, and of these 221 are very close wild relatives and 471 close wild relatives [25]. Thus, as a working estimate, there would be, globally, around 700 closely related CWR species (i.e., less than 0.26% of the world flora), which are of a high value in terms of their potential use in plant breeding programs and would deserve the highest priority to conserve the genetic diversity of major and minor food crops [21,28].

Vincent et al. [29] used the genepool and taxon group concepts to estimate CWR relatedness for 173 priority crops included in Groombridge and Jenkins [27] and the Annex 1 of the International Treaty for PGRFA. Additional taxa more remotely related to crops were added if they had useful traits for crop improvement. The inventory contains 1667 taxa, belonging to 1392 species in 108 genera and 37 families. It also includes ancillary data, such as their regional and national occurrence, seed storage behavior, and herbaria, housing major collections of CWRs. This inventory is available online as searchable resource, called the Harlan and de Wet inventory, and is actively maintained [30]. This list can be regarded as the most comprehensive one, based on clear criteria. A number of other global priority lists, typically developed in the context of specific projects, are less comprehensive, have less well defined or complex criteria, and have not been used as widely as the list by Vincent et al. [29]. Two African regional checklists [31,32] and several national checklists and inventories have also been developed and are available on the CWR global portal [33].

## 3. General Threat Status of CWR

Since the successes of the so-called Green Revolution in the sixties and seventies of the last century, with the breeding of high-yielding varieties of a number of important food crops worldwide, in particular by the CGIAR research centers, a vast replacement of traditional varieties of these crops by the newly bred varieties resulted in a significant loss of genetic diversity and triggered a systematic collecting and conservation of in particular landraces in the newly established genebanks. The Green Revolution also impacted on the agricultural production systems through the promotion of fertilizers and the use of pesticides, leading to a much more intensive agriculture. This development impacted also indirectly on CWRs, especially those that grew in cultivated fields, on field margins and along roadsides. Consequently, they were included in the global collecting efforts coordinated by IBPGR [9]. The authors reported that 25% of the collecting missions were dedicated to CWRs. About 60,000, or 27%, of the 220,000 collected samples were CWRs, mostly forages, including forage shrubs and trees (53.2%), followed by wild cereals (10.4%), wild legumes (9.4%), wild vegetables (7.6%), and wild root and tuber species (7.6%).

As for other wild plant species, the genetic diversity of CWRs continues to be eroded by global threats, such as: changing land use; climate change and natural calamities, becoming possibly the biggest threat through different specific impacts on CWRs; changes in agricultural practices; over-exploitation or excessive use; nitrogen deposition; and invasive species. Other factors include overgrazing and desertification; agricultural subsidies, such as that of biofuel crops, maize, and rubber; the development of aquaculture; reclamation of wasteland; pollution; and others [22].

Specific examples of global threats leading to genetic erosion of CWR species have been presented by [22,34] and [9]. The latter authors noted much fewer publications on genetic erosion of wild plants and CWRs, compared to those on crop species. Jarvis et al. [35] predicted a loss of almost half of the current geographic ranges of CWRs of peanuts in South America, cowpeas in Africa, and wild potatoes in Central and South America. They also projected that between 16% and 22% of these species would go extinct by 2055. Lira et al. [36] concluded from model studies in Mexico that eight of the wild Cucurbitaceae taxa will not survive under accepted climate change models. Erosion of traditional crops and their wild relatives is greatest in cereals, followed by vegetables, fruits, and nuts and food legumes [15]. As part of the GPA II implementation assessment for the period 2012–2014, 32 countries reported to FAO to have conducted more than 5200 PGRFA surveys, covering 1823 species (predominantly wild). Of these, 56.3% were rated as threatened, i.e., they were no longer cultivated or did no longer occur in situ in most of their previous areas of cultivation or occurrence [22].

The most commonly applied means of assessing threats to wild taxa are The IUCN Red List of Threatened Species criteria [37], including for CWRs [38]. Some countries, e.g., Germany, have their own system for assessing endangerment status at national level [39]. IUCN has started to place some focus on CWR threat assessments. Their Plants for People Initiative, for example, included the assessment of high priority CWRs. CWRs are flagged within the IUCN Species Information System. The IUCN Red List of Threatened Species version 2017-2 included 760 CWR assessments [40]. The IUCN Red List status was assessed for 572 CWR species in Europe, and 11.5% of these species were classified as threatened (categories ‘vulnerable’, ‘endangered’, or ‘critically endangered’) and 26 species were reported as ‘near threatened’ [41]. Bolivia established a red list of CWRs using the IUCN criteria [42]. Maxted et al. [28] reported that the loss of genetic diversity within CWR species is likely to be much greater than the loss of species. Most of the species that are able to survive the threats they are exposed to will suffer some genetic erosion or loss of genetic diversity. The increasing impact of climate change is likely to impose heavy selection pressure on CWR populations. This could easily lead to a loss of genetic diversity and, consequently, species may not be able to adapt as readily and quickly to changing conditions as before. Thus, this vital diversity that is required to underpin food security might not be any more available to breeders [28].

Genetic erosion occurs also in genebanks due to intercrossing with other accessions during regeneration, selection, genetic drift, and shift because of unsuitable growing conditions, loss of viability in storage, or also due to human errors during cultivation. As CWRs are difficult to grow, genebanks might tend to wait as long as possible with regenerating them and, thus, seeds might lose their viability and thus cause genetic erosion [43]. The lack of knowledge about the biology of CWR species, the absence of a good infrastructure for their cultivation, and other factors, such as adequate funding for conservation, might well contribute further to genetic erosion, in particular within accessions [9].

## 4. An Assessment of Peculiarities of CWRs with Respect to Conservation Management

### 4.1. Biological Peculiarities

CWR species possess characteristics that allow them to survive in nature. Such characteristics are, in many instances, not suitable for cultivation. As CWRs are most valued and valuable as reservoirs of new genetic diversity and traits required by plant breeders, this diversity is evolving in nature while being exposed and adapting to (changing) environmental conditions. Storage in a genebank would not allow such adaptation processes to take place while being conserved. This means that one has to consider where to conserve the CWR, i.e., in their natural habitat (i.e., in situ), in a genebank or botanic garden (i.e., ex situ), and/or a combination of the two. Both the GPA II [16] and the CBD [15] regard in situ conservation as the strategy of choice for CWRs, backed by ex situ.

With respect to in situ conservation, the obvious advantages compared to ex situ conservation are that CWRs can be conserved dynamically, providing for ongoing evolution and for a wider coverage of their genetic diversity. However, a number of preconditions to achieve this are presently not met, including lack of biological information on the species themselves, their taxonomy, distribution, and threat status.

With respect to ex situ conservation, one should realize that crop species have lost most or all of the ‘wild’ characteristics during the domestication process. Typical examples are shattering, day length sensitivity, variable and non-determined flowering period, fragile ears (in the case of cereals), etc., which CWRs do possess. Thus, their management in an ex situ condition might be very difficult and requires ample experience. Many wild species have a limited distribution area, compared to most crops, and are an integral part of ‘their’ natural ecosystem. Their adaptability might be limited and, thus, also their ability to adapt to new environments (i.e., in particular, those of a genebank setting) might be low. Consequently, their optimum ecological conditions should be known when growing them outside their distribution area, in order to produce healthy and vigorous seeds/planting materials for subsequent storage. Furthermore, their biological reproduction ‘system’ should be known to ensure an effective reproduction, especially in case pollinators are required.

Storage behavior of CWR seeds might be unknown as seed biological aspects are unknown and, thus, require testing to ensure optimum storage conditions; standard viability seed testing methods might not function properly and/or more advanced viability tests might be used; collected seeds might be very variable in quality, i.e., not uniform in their maturation status and, thus, with variable longevity expectations; seeds might have dormancy and/or could possess hard seeds, whereas no treatments are (yet) known; typically only small samples have been collected and, thus, there is in general a need for (immediate) multiplication before storage; possible presence of pest and disease in or on the material (vegetative material, non-orthodox seeds, and/or orthodox seeds) could have implications for outgrowing in the field or greenhouse, for viability testing, storage, and distribution [44].

The lack of knowledge and information on the existence, distribution, and genetic diversity patterns of CWRs make their adequate collecting difficult. This includes the application of the best possible sampling strategy, including the number of plants per population (if there would be such an option to decide), the number of populations for a defined area, or even the entire distribution area of a given CWR, the right timing of the collecting mission, etc. (for details of these and other collecting aspects see [45,46,47]). This general lack of information is certainly one of the main reasons why CWR genetic diversity is sub-optimally represented in ex situ collections.

Notwithstanding the high importance accorded to in situ conservation of CWR, in particular in protected areas [21], the effectiveness is reported to be more uncertain than in genebanks. At the same time it should be noted that the main rationale for in situ conservation is based on the likelihood that continued exposure to changing selective forces will generate and favor new genetic variation and, thus, there is an increased chance that rare alleles that may be of value to future agriculture are maintained [48].

In addition, considering the rather huge numbers of CWR species reported (50,000–60,000 species), the need to conserve adequate representation of selected populations for each CWR species is creating big challenges for an efficient conservation of CWR diversity [28].

### 4.2. Managerial Responsibility- and Awareness-Related Issues

It should be realized when establishing priorities for CWR conservation that their natural distribution does not follow, in most instances, national borders. Consequently, consultations with neighboring countries could facilitate comprehensive and effective conservation of the entire CWR genepool. In addition, information on the spread and possible distribution patterns of the genetic diversity within a given CWR genepool will be very helpful to identify possible sites for in situ conservation and/or to apply the most efficient sampling strategy when collecting.

According to the CBD, the CWR occurrences are under the sovereignty of the countries in which they grow. Therefore, in situ conservation of these species has, necessarily, to include a strong national component and any regional or global in situ conservation approach should be based on and/or aim to integrate or complement such national and local in situ actions. CWR in situ conservation cannot be centralized at national or international level, as is possible with ex situ conservation in genebanks.

According to FAO [21,22], in many countries, CWRs do ‘fall between the cracks’ of the responsibilities of the environmental and agricultural sectors. This makes it difficult to decide which organizational entity should be the ‘logical’ institution to assume the conservation responsibility in a given country. Constraints related to this decision are the fact that CWRs are still a not sufficiently known genetic resource, that they have been knowingly or unknowingly included in nature protection measures without specific management or monitoring activities [28,48], and that they have been maintained by botanic gardens or genebanks without communication with other stakeholders.

Due to the disadvantaged position of CWRs compared to the domesticated genetic resources in most countries, the public awareness on CWRs is, in general, very low; there is no or only a weak political lobby within institutes and countries and, thus, a low priority to apply or provide funding for their conservation. Furthermore, there is a need for training and capacity building; skills such as taxonomy are limited and dwindling, creating dependencies on other organizations and countries. Especially in (remote) rural areas, there is a big need for better awareness and appreciation of CWRs, their diversity, and their role in breeding and adaptation to climate change for sustainable agriculture in order to stand any change of creating sustainable conservation initiatives.

The establishment and operation of in situ conservation sites can present administrative, logistical, and legal problems. For instance, CWR species that occur in ‘disturbed’ habitats, such as road-sides and field margins, as well as abandoned agricultural areas, will most likely not be ‘included’ in a protected area [28] and, thus, will require either their ‘own’ in situ conservation efforts, for instance, as part of an on-farm management scheme, and/or should be included in ex situ conservation. However, in many instances, their existence might not be known to the national PGRFA programs and/or the local authorities or conservation projects and, thus, are not on anybody’s radar.

When considering the conservation of CWRs in protected areas, it should be noted that this type of in situ conservation is likely passive, meaning that CWR populations located in protected areas are not being actively managed and monitored, as most of the protected areas that harbor CWR species do not have specific CWR management plans [25]. Active and effective conservation of CWR populations located in protected areas could be achieved by expanding the management plans by including specific actions targeted to CWR [16]. Furthermore, climate change might lead to pronounced range contractions or range shifts for many CWRs. This led Aguirre-Gutiérrez et al. [49] to investigate the impact of climate change on CWRs and to combine this with monitoring programs, as well as collecting of CWRs for backing up in ex situ conditions. They conclude that in situ conservation measures, when ignoring the effects of climate change, will not be effective for many CWR species and that large-scale ex situ conservation actions are needed to safeguard CWRs.

CWRs can create problems for genebanks to manage them in routine operations, in particular, when specific required species information is lacking. For instance, to regenerate or multiply CWR accessions in the field or green or screen house, a genebank manager has to cultivate these wild species and, therefore, has to find answers to manage characteristics, such as a possible low germination rate, the unknown reproductive biology of the species, possibility of small sample sizes, shattering, non-homogenous ripening, etc., in order to meet the agreed standards for genebanks [50,51]. The lack of knowledge, experience, and facilities to adequately manage CWRs in genebanks is widely recognized. Thus, many genebanks will have to seek collaboration with other scientists in the country or with other genebanks that have more expertise in conserving CWRs. One option could be participation in a regional CWR network, through which the coordination of activities with neighboring countries could be achieved, sharing of responsibilities could be obtained, etc. The European Cooperative Program for Plant Genetic Resources (ECPGR) and its virtual European genebank, AEGIS, is an example of such a regional network [52]. At the same time, it should be noted that the conservation of CWRs only ex situ would not be feasible because of the sheer number of species and the need to sample and conserve eco-geographically and genetically diverse populations for each species in a dynamic way [28].

### 4.3. The Need for Prioritization of CWR Taxa

Considering the large numbers of species that are classified as CWRs, the usually limited financial resources for conservation, and the fact that many CWR species are not well known and in most cases lack critical information, there is a strong need to set clear priorities for their effective conservation. Possible prioritization criteria for CWRs should address aspects such as:

1. the degree of threat of the species;
2. their genetic closeness to the crop species;
3. the demand for specific traits/species by the (potential) users (and thus their economic potential);
4. the distribution area (uniqueness, incl. endemism; centre of origin/diversity) and occurrence of a given species;
5. the conservation status of a given species, including in other (neighboring) countries of the distribution area;
6. the (physical as well as legal) availability; and
7. the international legal and policy instruments vis-à-vis the national legal framework.

These criteria are based on priority-setting criteria that have been used and reported in [53,54,55,56]. When countries need to prioritize CWR species they will select a number of these criteria in accordance with their national context. The choice and assigned importance of criteria are therefore likely to vary between countries, while the most commonly included criteria are the economic importance of the related crop, the genetic closeness to the crop, and the threat status of the CWR.

Whereas priority-setting is a ‘standard requirement’ in conservation, both for in situ as well as for ex situ approaches, there are some specific impediments to the prioritization process of CWRs. Possibly the most important factor is the lack of information/knowledge on the species themselves (see also the following section). Another important constraint is that CWRs are typically not ‘directly’ used and, thus, not part of a traditional ‘food system’ (and consequently of a traditional knowledge system) or of an agricultural production system and, thus, their intrinsic value is often not recognized.

### 4.4. Availability of and Access to Data and Information

Availability of and access to data and information about CWRs, i.e., their occurrences, distribution, and threat status, their taxonomy, biological characteristics, ecological requirements, habitats, uses and genetic and phenotypic characterization and evaluation, are essential for the planning and implementation of effective conservation and use of CWRs. Existing information is yet mostly scattered, held in different formats (including non-digital) by very disparate entities, many outside the PGR community, and often not readily available. In hardly any data source, CWRs are flagged or tagged as such. Accessing this information is, therefore, resource intensive and time consuming, even more so as comparing datasets is often very difficult due to the variety of standards, formats, and data management models used [26,57,58,59]. However, quite some progress in proposing descriptors and data collection formats has been made in the past few years, e.g., [26,60,61,62,63,64]. In addition, data are often incomplete and new and/or more data need to be generated or collected. For example, data about occurrences of CWR populations are usually derived from databases of ex situ genebank accessions and herbaria specimen records. These most often do not reflect a comprehensive picture of the species’ distribution, can include very old records, and do not include data about the population status of the recorded occurrence. Field surveys and collecting require solid taxonomic knowledge of the local flora, which can be difficult to source. A global database or catalogue that collects into one place data about CWR inventories, occurrences, distribution, and in situ conservation actions currently does not exist.

## 5. The Current CWR Conservation and Use Status

### 5.1. Facts and Figures on CWR Conservation

#### 5.1.1. In Situ Conservation

Whereas the CBD [14], as well as the GPA [16], recognize the importance of CWR in situ conservation and regard ex situ conservation as a complementary conservation effort, the progress of CWR in situ conservation remains slow and difficult. In the second State of the World (SOW II) report, it is noted that in situ conservation is often envisaged to take place in protected areas or habitats and can be targeted at the species or at the ecosystem in which they occur [21]. However, the report also noted that in situ conservation of wild species of agricultural importance occurs mainly as an unplanned result of efforts to protect particular habitats or charismatic species. Furthermore, existing in situ protected areas do not always meet the required management standards to maintain CWR populations and their genetic diversity long-term [65]. Whereas the number of protected areas globally has increased considerably and the total area covered by protection expanded from 13 in 1996 to 20.3 million square kilometers in 2020, covering 15.2% of the terrestrial surface [66], it should also be mentioned that, in general, areas with the greatest diversity, for instance within centers of origin and/or diversity of our crops, have received significantly less protection than the global average [21].

Several countries informed as part of the SOW II report [21] the establishment of protected areas for CWRs, e.g., Armenia (CWRs of cereals), Ethiopia (wild populations of *Coffea arabica*), Mexico (*Zea perennis* and *Z. diploperennis*, CWR species of maize), China (86 in situ conservation sites for CWRs of different crops), Turkey (protected areas for CWRs of cereals and legumes), and Syria (protected areas for CWRs of cereals, legumes, and fruit trees). Hunter and Heywood [55] reported the establishment of a citrus wild relatives’ gene sanctuary in northeast India in 1981. A similar genetic reserve for wild relatives, including relatives of lychee, longan, and citrus, was established in Vietnam. They also mentioned that certain wild species of mangoes and other wild relatives are known to occur in biosphere reserves, national parks, and other reserves in India, Indonesia, Singapore, the Philippines, Thailand, and Sri Lanka, but little targeted in situ conservation has been undertaken. In Europe, the first CWR genetic reserves were designated in 2019, when, in Germany, a network of genetic reserves for four wild celery species was established [67,68,69]. As of February 2020, the network included 15 genetic reserves and more are in the process of being established.

The aforementioned summary assessment of GPA II [22] noted an increased attention to CWRs in the context of in situ conservation and management. Overall, 14.2% of the over 15,000 in situ conservation sites that were listed in 20 country reports had management plans addressing CWRs and wild food plants. A total of 78 activities on in situ conservation and management were implemented with institutional support in 19 countries. A total of 16 countries reported an estimated total of 2141 CWRs, including species from primary and secondary genepools, as well as species previously used for breeding but belonging to the tertiary genepools, and wild food plants, actively conserved in in situ areas. The average per country is amounting to 134 CWR species with a maximum of 840 species in one country. However, the overall developments, with respect to the implementation of the in situ conservation priority activities of GPA II, were limited in scope and the reporting countries rated their achievements with respect to this priority activity as the lowest across all the 18 priority areas that make up the Second GPA [22].

Vincent et al. [65] assessed 167 of the most important food crops for improving food security and income generation and identified 1425 priority CWR species related to these crops. They modeled the distributions of 791 of these priority CWRs as the basis for the identification of 150 sites for in situ conservation. Individual CWR species, in general, were found to be well represented in current protected areas; only 35 (2.5%) of the studied species, related to 28 crops, were distributed exclusively outside of protected areas. If a threshold of 50% or more of the potential genetic diversity of a CWR, based on ecogeographic land characterization diversity [70], occurring within protected areas, is considered adequate for genetic conservation, then 112 of the assessed CWR species are under-conserved, while 91% of CWRs are well represented within existing protected areas. Effectively conserving the top 10 CWR sites inside protected areas and the top 10 sites outside protected areas as defined in the pragmatic scenario, would only require active management of ~2000 km^2^ globally and would protect 475 CWR species, and 1257 unique CWR/adaptive scenario combinations. Vincent et al. [65] propose to manage these as a global in situ conservation network.

As any other wild species, most of the CWRs might not have any direct economic or nutritional relevance to local communities and, thus, might not be of interest to them. In fact, some of them might even be weedy and constitute a nuisance to local farmers. Therefore, CWRs might not be very attractive for inclusion in a local ‘on farm’ conservation program [15], and in case their distribution area is not part of a protected area setting, local communities will not be interested in participating in a conservation activity if no benefits/funding will be provided. Only in cases where the CWR species occur in a protected area (targeted or ‘by chance’: [15]), their conservation might be easier and more sustainable as long as some sort of a monitoring system does exist.

In some cases, however, CWRs play a known and appreciated role in local and, typically, traditional cropping systems and, thus, will be valued by local farmers or communities. Consequently, conservation approaches might be easier and could directly involve the local people, as long as benefits will be generated through such activities. Examples of such situations include the regular re-domestication of *Dioscorea cayensis* subsp. *rotundata* in Benin; the use of *Dioscorea* spp. in West African countries by facilitating the introgression between wild and domesticated yams, as this is an important improvement strategy; the use of *Ensete ventricosum* in Ethiopia for regular incorporation of ‘wild’ seedlings into the fields of the cultivated crop; or the selection of wild walnut genotypes for cultivation in Kyrgyzstan [21]. From a crop evolutionary perspective and more related to traditional agricultural production systems, tolerating CWR species which are weeds, especially in field-borders, as pollinators of the cultivated material and, thus, assumingly increasing the genetic diversity of the crop for subsequent selection, is another example. However, also the opposite can be true that CWR play a detrimental role in farmers’ field, for instance, as noxious weeds.

#### 5.1.2. Ex Situ Conservation

Traditionally, ex situ conservation is the main approach that countries have taken to conserve CWRs. Genebanks play an important role in the overall conservation of CWR germplasm; in fact, they (should) provide a link between in situ conservation and the users’ communities at various levels. This role is essential as they typically are specialized in long-term conservation, distributing or exchanging requested materials, characterizing and evaluating the stored accessions, keeping detailed information records on the individual accessions and, in some instances, conducting pre-breeding activities to facilitate the use.

Genesys, the largest global database on ex situ conserved germplasm accessions, provided data (as of 11.01.2020) for 4,097,112 accessions, of which only 12% are classified as wild material, thus possibly also including some non-CWR species [71]. The European Search Catalogue for Plant Genetic Resources (EURISCO) [72] contains data for 2,023,530 accessions. Among those, 12.15% are reported as wild. According to Ford-Lloyd et al. [34], the 1095 CWR species reported in EURISCO, at the time the research was undertaken, only represented 6% of the 17,495 CWR species found in Europe. This means that 94% of European CWR species are not conserved in ex situ collections.

The SOW II report [21] provides an average percentage of wild species, predominantly CWRs, for each of the 11 major crop groups, varying from 4% (food legumes and fiber crops) to 35% (forages) and 46% (industrial and ornamental plants). The overall mean for the almost 7 million reported accessions of wild plants is 10%, most of them being CWRs.

For a number of reasons, many CWRs are represented by a small number of accessions per species in the collections, both in genebanks and in botanic gardens. As an example, of the 1076 global priority CWR taxa identified in a study about global CWR conservation priorities [73], ‘only’ 763 or 70.9% are included in genebanks; among those, 257 taxa are represented by less than 10 accessions each. Over 95% of the taxa examined were found to be insufficiently represented in genebank collections with respect to their full range of geographic and ecological variation in their native distribution area. In many instances one would find just few accessions per taxon, e.g., only 5.4% of the CWR taxa in EURISCO are represented by 10 or more accessions, whereas 90.5% of the CWR taxa have less than 5 accessions.

Due to the already mentioned difficulty to collect adequately sized numbers of seeds/plants per population, many of the accessions consist of (too) small quantities of seeds and are genetically poorly sampled [74]. In addition, the stored seed samples have frequently a low(er) viability due to the difficulties to grow them out for regeneration purposes [75]. Another aspect, related to lack of information/knowledge, concerns taxonomic identification of the CWR, including to which crop genepool they belong. This will directly impact on the priority-setting and possible subsequent conservation, both in situ and ex situ, as well as on their use.

**Figure 1:**
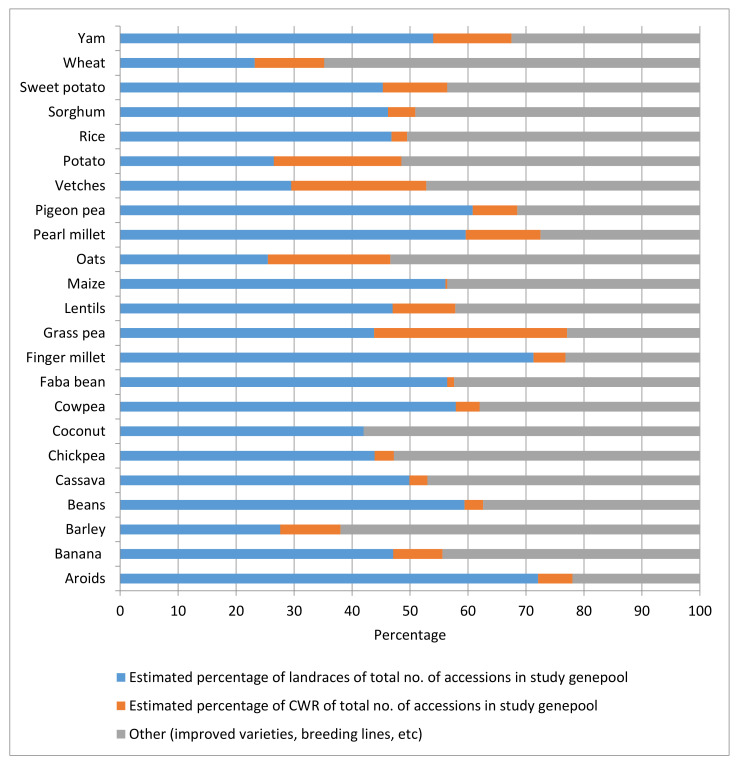
Percentages of CWR and landrace accessions in genebank collections of 23 selected crop genepools.

In a study of ex situ holdings of 23 selected genepools of the major crops included in Annex I of the International Treaty, i.e., those materials that countries agreed to form the backbone of the multilateral system of the International Treaty, the authors calculated an average non-weighted percentage of CWR accessions in genebank collections (without the international collections held by the CGIAR genebanks) of the selected genepool worldwide of 9.6%, ranging from 0% for coconuts (there are no CWRs known) to 33% for grass pea (a little bred crop) (Figure 1). The total global holdings considered in the study of the selected genepools (without the collections held by the CGIAR genebanks) were 3,149,371 accessions [76].

When looking at the primary genepool, 242 of the 1667 CWR taxa included in the Harlan and de Wet CWRs inventory were found to be under-represented in ex situ collections and the countries identified as the highest priority for further germplasm collecting are China, Mexico, and Brazil [29]. Khoury et al. [77] used gap analysis to assess the degree of representation of *Cucurbita* CWR taxa in conservation in situ, as well as ex situ in genebanks and botanic gardens. For the *Cucurbita* genus, including 16 CWR and six cultivated species, the authors established detailed taxon-related ex situ, as well as in situ (i.e., protected areas) conservation priorities and suggested further in situ protected areas that would cover the greatest amount of populations of the largest number of taxa. Khoury et al. [77] concluded that 68.8% of wild *Cucurbita* taxa were assessed as high or medium priority for further collecting for ex situ conservation and 81.3% had a high or medium priority for further protection in situ, including all of the progenitors of the cultivated species. Furthermore, four taxa were listed as having very few accessions and, thus, very limited diversity is available for crop breeding. Khoury et al. [77] suggested that these figures might be considered as ‘typical’ for the CWRs at large.

Besides their conservation in situ and in genebanks, botanic gardens have also been collecting and storing CWR materials in their collections, as demonstrated by the PlantSearch database, which is an information platform for 1155 botanic gardens that collectively maintain plant, seed, or tissue collections of 589,526 taxa [78]. The database reveals that botanic gardens maintain at least 30% of all known plant species in their own collections, including that more than 41% of species assessed are globally threatened. Many of these wild species are CWRs. Almost one-third (315, or 28.6%) of the 1076 aforementioned global priority CWR taxa are maintained by botanic gardens [79].

A recent major effort of collecting new CWR samples was made by the project “Adapting Agriculture to Climate Change” [80], which focuses on the wild relatives of 29 crops included in Annex 1 of the International Treaty; over 4500 new CWR samples were collected for ex situ storage, evaluated for useful traits, and enhanced or pre-bred for use in crop improvement programs.

#### 5.1.3. Complementary Conservation

As already noted above, both the CBD [15] and GPA II [16] refer to the need to complement in situ conservation efforts with ex situ measures. Genebanks have recognized strengths in facilitating easy and targeted access to specific material (which is problematic for in situ conserved material) and to allow secure and long-term conservation as part of the conservation and use continuum. Especially when environmental change is too rapid for evolutionary change and adaptation, or migration, it can be easily understood how and why ex situ measures would complement or even replace in situ conservation and thus provide for the most effective approach [22,81]. Such a complementary approach requires that in situ and ex situ conservation measures have to be carefully planned and combined, thus securing a holistic combination of the two, which capitalizes on strengths and avoids weaknesses of one or the other. This will require a good understanding of the (seed) biology of the species, their threat status, priorities assigned to the individual CWR species, and other aspects; an assignment of clear responsibilities, including, for instance, to the agricultural and environmental sectors; if applicable, to link conservation and development; adequate and comprehensive information management; facilitation of adequate coordination with other stakeholders and countries; the verification of clear ownership rights over areas where the to-be-conserved CWRs occur; support of public awareness on the importance of CWR conservation; and, where necessary, to ensure the engagement of the broader public.

As an example, Hunter and Changtragoon [82] conclude, on the basis of regional project experiences, that for wild relatives of tropical fruit trees, any conservation strategy should contain elements of both in situ and ex situ conservation and should have a focus on conservation, both inside and outside protected areas. It should also ensure coordination of planning and implementation, institutionalize the practice of wild relative conservation, promote public awareness and understanding, create a suitable policy environment, and highlight the many benefits derived from their sustainable conservation and use. In situ approaches seem feasible for conserving wild relatives of tropical fruit trees, but experiences with targeted species and actions inside and outside protected areas appear to be relatively few. Consequently, wild relatives of tropical fruit trees remain a largely under-conserved natural resource, both ex situ and in situ, and are continuously under threat in their natural habitat from neglect and over-harvesting [82]. Vincent et al. [65] note the generally accepted requirement for complementary conservation, i.e., to also cover in situ conserved materials in genebanks, a process that has started recently. They further see a particular need to develop CWR in situ activities that enable the conservation of geographically partitioned genetic diversity which retains potential for local environmental-evolutionary adaptation.

### 5.2. Facts and Figures on CWR Use

The term ‘use’ needs to be applied in its widest sense for CWRs. The traditional understanding is the use of genetic diversity in plant breeding by crossing cultivated material, usually advanced varieties with CWRs and through a strong selection to obtain genotypes, with the traits that have been transferred from the CWR species. Furthermore, CWRs are an important target for research on crop evolution and are, indirectly, an important component of research on the origin and spread of agriculture. With the increasing focus of conserving CWR in situ (including on-farm), the ‘direct’ use of CWRs by local communities and farmers has now also received some more attention. Another dimension of ‘using’ CWRs is their not well understood and accepted role in and contributions to the evolution of crops and plants at large. Through the overall conservation efforts of the flora (and fauna) in natural habitats and protected areas, of which CWRs are an integral part, they contribute to a healthier environment, healthy ecosystems, and the provision of ecosystem services. However, this latter aspect is not part of the focus of this paper. Furthermore, the appreciation of the economic value of CWRs and their contribution to the global economy is an aspect that would fall under the term ‘use’.

In tropical zones, wild fruit harvested from forests contribute significantly to the total income and to sustainable nutritious diets of many rural households, apart from contributing substantially to important ecosystem services [29]. Wild relatives and wild-growing semi-domesticated species of tropical fruit trees also provide services to domesticated fruit trees in terms of resistance to extreme abiotic and biotic stresses through their high levels of genetic diversity [82].

More widely applied is the use of CWRs in pre-breeding and breeding programs and in research, in particular in countries with strong breeding companies, where facilities and technologies, as well as funding, are available to exploit these ‘difficult’ resources. Today, climate change is causing dramatic changes that are being experienced around the globe, especially global warming and the related increase of severe erratic weather conditions. These changes have a significant impact on agricultural production systems that need to be addressed as well. To allow crops to cope with and/or to adapt to more extreme weather conditions, including heat, drought, flooding, and increased salinity, there is a strong need for more genetic diversity than currently available for most crops from which plant breeders can select specific traits and resistance genes to ‘equip’ new varieties to cope with these changing conditions. In particular, the use of CWRs, as a known source of traits for introgression into the crops, has proven to offer such solutions, especially to overcome biotic stresses [8]. As CWRs do possess a much wider array of traits and allelic diversity, as well as ‘new’ genetic variation compared to our modern crops, they are an important asset to be included in the breeding pools of our plant breeders and, thus, to be accorded a high priority in their conservation and research and management activities that facilitate their use by plant breeders, worldwide [73,83,84].

‘Historical’ examples of CWRs in plant breeding include the use of wild *Aegilops*, *Secale*, *Haynaldia*, and *Agropyron* species in wheat breeding [85], the introduction of resistance to late blight, which is caused by *Phytophthora infestans* and is found in the wild potato *Solanum demissum* [86], as well as other disease resistances and tolerances from different potato CWRs [87]. Resistance against stem rust caused by *Puccinia graminis* subsp. *graminis* derived from the wild wheat *Aegilops tauschii* [88], in another example. In the early 1970’s, resistance to the grassy stunt virus was found in wild *Oryza nivara* and now this gene can be found in almost all material bred by the International Rice Research Institute in the Philippines [34]. Maxted and Kell [25] reviewed the use of CWR in crop improvement in 291 papers reporting the identification and transfer of useful traits from 185 CWR taxa into 29 crop species. Wheat and rice accounted for almost 84% of the transfers and 56% of the inter-specific trait transfers related to pest and disease resistances.

The above historical examples demonstrate the past focus on trying to identify traits of interest through phenotypic characterization and evaluation [28]. Whereas the inclusion of genetic diversity from the wild genepool in breeding activities was difficult [21], the advancements in molecular genetics and the related tools allow a much more ‘targeted’ use of CWRs. Through the possibility of transferring specific parts of the genome, i.e., traits, genes, and/or alleles into the genetic background of improved breeding materials, the hesitation of using CWRs is fading and, thus, their importance for breeding is increasing. According to Ford-Lloyd et al. [34], genomic-based resources, map-based cloning, analysis of quantitative trait loci, gene isolation, and genetic modification are increasingly significant to exploit the potential of CWRs. Genomic databases containing information on genes associated with adaptive characters must increasingly be linked to web-enabled databases of ex situ conserved CWR germplasm, such as EURISCO [72]. Furthermore, predictive characterization, Focused Identification of Germplasm Strategy (FIGS) [28] and eco-geographical filtering method [89] are other promising approaches to facilitate the use of CWRs in breeding.

The number of CWR genomes sequenced has grown significantly over the past decade and in 2016 the number of crop genomes sequenced was ‘only’ about three times higher than that of sequenced CWR species, which were about 40 [90]. For example, Bertioli et al. [91] sequenced two wild peanut species (*Arachis ipaensis* and *A. duranensis*). Peanut is an important food source for many farmers in the developing world. The CWR genome sequences will provide breeders with new tools for enhancing the crop, and for developing new varieties more resistant to pests, diseases or with improved abiotic tolerance traits. It is hoped that this positive trend of more CWRs to be sequenced continues and thus, allows a better exploitation of the important traits that CWRs harbor, including quantitatively inherited traits.

A study carried out by PwC [92] assigned an indicative value of $42 billion to the CWRs of 29 major food crops, with a potential to reach a value of $120 billion in the future. All these 29 crops are included in Annex 1 of the International Treaty on PGRFA. Pimentel et al. [93] reported an estimated value of $115 billion that CWRs contributed toward increased crop yields per year worldwide. In addition to their economic value, CWRs are also being valued for their not so well-known contributions to ecosystem services [34]. Tyack and Dempewolf [94] have reviewed past economic values of CWRs, including the previously cited studies, and propose an improved conceptual model for understanding the economic value of CWRs under climate change, expanding it from the focus of gross production to including a series of other values and costs.

## 6. What Needs to Be Done to Conserve and Use CWRs More Effectively?

From the information, facts and figures presented above, it is apparent that further concerted assessment and conservation efforts are required in order to keep these valuable resources and the traits therein available and accessible to the users, now and in the future. In this section, we summarize findings and identify actions for efficient conservation and sustainable use of priority CWRs. Important aspects that require attention to underpin the conservation efforts are presented.

### 6.1. Documentation

Documentation and availability of CWR data are the basis for the assessments of conservation and threat status, conservation planning, and monitoring, but are yet insufficient to provide more precise assessments and concrete figures about status and trends of CWR diversity. In recent years, tools and descriptors have been developed to support CWR data collection and management (see Supplementary Materials Table S1). The Secretariat of the International Treaty is currently developing a globally agreed descriptor list for CWR data exchange as a further step towards harmonizing CWR data recording and exchange and facilitating the development of national and global CWR databases. Based on these standards and tools, all relevant data at national level required for CWR conservation planning and management should be brought together in an accessible as well as standardized format into national CWR databases or portals. Furthermore, the development of a global CWR data portal, analogue to Genesys, the global hub for ex situ data, should be considered. National CWR databases could then provide data to this global resource. Such a global portal would allow reaching a better understanding of global CWR distribution and conservation status. It would serve as an important tool for sharing information and supporting more effective planning, conservation, and monitoring at the national and international levels, as well as international collaboration in CWR conservation.

An increased recognition among the actors within the environmental sector responsible for nature protection and protected area management that CWRs constitute a group of very valuable PGRFA, would possibly support flagging and data recording in their respective databases and monitoring activities, and integration of CWR conservation aspects into existing nature protection networks and activities.

### 6.2. In Situ Conservation

As each country is responsible for the conservation of the natural resources within its territory, CWR conservation is logically and mainly addressed at national level. To secure these resources effectively and long-term, systematic and coordinated conservation is essential, as well as integrating in situ and ex situ measures. In most occasions, however, CWR in situ conservation has been carried out within the framework of projects, which are limited in time, hardly ever running for more than five years. A more stable organizational and financial basis for CWR conservation at the national level is therefore required in most countries. This can be supported and facilitated by developing a national strategic action plan for CWR conservation.

There is no single method for planning CWR conservation or for developing such a strategic plan, as related factors, such as financial and human resources, availability and quality of baseline data, the range, role and responsibility of relevant stakeholders, or the commitment of national governments, vary between countries. Nevertheless, a series of steps and decisions in the conservation planning process are likely to be common in most situations. These include the development of a CWR checklist, prioritization of CWRs, development of an inventory of the priority CWRs, threat assessments, gap and diversity analyses, and the identification of priority sites and actions for in situ and ex situ conservation [56].

The development of a national CWR organizational plan and an efficient coordination mechanism are important to facilitate coordination and collaboration. These measures require and will greatly benefit from the establishment or provision of a nation-wide information platform that facilitates the routine operations, allows the necessary coordination, and enables adequate reporting. The collaboration between the various important stakeholders at the local, provincial, and national levels is a prerequisite for effective and sustainable conservation operations. At the national level, adequate coordination between, in particular, the ministries of agriculture and environment and their implementation bodies is critically important to facilitate the identification and management of protected areas that target or include CWRs and to allow the participation of key stakeholders in the planning and implementation of projects and activities, including the support of research and awareness creation. Considering the specialized skills and facilities required for efficient and effective conservation of the CWR genepools, close collaboration with neighboring countries, possibly in the context of a regional network, seems to be very important to allow an adequate conservation of the total genetic diversity range of a given CWR species.

### 6.3. Ex Situ Conservation

Targeted and adequate collecting of highly threatened and prioritized CWR materials from their natural distribution areas, as well as of populations that are requested for research and use, is a critically important step to avoid genetic erosion and to facilitate use. A close collaboration with local communities and their conservation activities is important, as well as coordination with botanical gardens and other ex situ conservation programs. During collecting, it is important that an adequate number of populations of targeted CWR species is sampled and that the samples are of an adequate size. To ensure effective conservation for each collected CWR species, specific conservation standards need to be used; where necessary, further research might be required. One such research area is on seed biological aspects (see, for instance, [44,51] and/or the application of already developed advanced methods, e.g., on germination testing, using potential markers as volatile compound [95,96], changes in methylation [97,98], or DNA and RNA integrity [99,100]). The morphological and/or molecular characterization as well as further evaluation of conserved samples will be an essential step to facilitate their use, where applicable this should be done in collaboration with neighboring countries. One other example could be the application of cryopreservation of embryos, cells, tissues, or seeds as a long-term conservation method, especially for CWRs that cannot be conserved in the form of orthodox seeds.

A national CWR priority list provides the foundation for targeted collecting of threatened populations and for the development of complementary conservation efforts that reflect the long-term conservation needs, the biology of the species, the needs of users, accessibility to specific materials, and the requirement of exchanging/distributing germplasm. Well planned characterization and evaluation of prioritized accessions will increase our required knowledge and understanding of the genetic diversity aspects of the CWRs and thus enable and facilitate effective conservation as well as the targeted and sustainable use of conserved material.

### 6.4. Complementary Conservation Approaches

When planning CWR conservation approaches, a number of considerations will be important to take into account, especially when realizing that in general limited information is available about these resources. Furthermore, different infrastructures and technologies are needed to collect, conserve and monitor the material under conservation. In addition, geographical, technological, scientific as well as political/legal aspects will have to be considered and should complement each other well. As mentioned before, complementary conservation is not a ‘method’, but rather a conceptual framework that helps with the systematic planning of conservation efforts for a given species and under specific ‘local conditions’. An example of such a framework is provided in [101]. So far, little practical experience can be reported. The approach should lead to practical and efficient, long-lasting, and cost-effective conservation activities for a given species. Examples of such pragmatic approaches would be to include populations of CWR species conserved in situ also in ex situ storage as a safety back-up and to facilitate their access for use. In case species cannot be (safely) conserved in situ, for instance, due to financial or administrative constraints or when the species is highly threatened, attempts should be made to conserve the threatened species ex situ in a genebank.

As use might be regarded as the ultimate goal of a conservation effort, it seems obvious to involve the users (primarily breeders) also in a prioritization and conservation planning exercise. Thus, the requirements of possible users of conserved germplasm can be duly reflected in the conservation approach, including specific aspects such as that the conserved materials can be shared easily with users in an appropriate form and quantity.

The very fact that only limited practical experience has been made with complementary conservation, the fact that the best possible combinations will vary from place to place and species to species, means that it will require more research to allow optimal solutions for effective and efficient conservation and sustainable use of individual CWR species to be identified. The development of a generic decision tree and supporting guidelines could be an important contribution to a more comprehensive, effective, and efficient complementary conservation of CWRs, at the various levels.

### 6.5. Supporting Use

Concerted efforts that facilitate the use of conserved CWR germplasm, either in in situ or ex situ conditions, are needed to enable a more effective and increased use of the often-unique genetic diversity contained in these threatened resources. Such efforts can be very diverse and include for example better management practices in a genebank or protected area, with respect to the representation of genetic diversity (as populations and/or as pure lines, etc.), ensuring an adequate coverage of the genetic diversity that exists within a species in the collection, and very importantly increasing the level of characterization and evaluation of individual accessions (both morphological and molecular), providing much more information on the CWRs conserved in genebanks and improving the availability and accessibility of data.

### 6.6. Strengthening the Conservation System

In the context of this paper, the national approach is possibly the most relevant one, but with the clear understanding that the ‘real action’ will have to be undertaken ‘on the ground’ at the local level and, whenever possible, for both in situ and ex situ approaches. However, when considering the many difficulties to ensure an effective and secured conservation of these species, it is obvious that many of the less well-endowed local genebanks and botanic gardens will require support to implement such conservation activities adequately, in order to contribute to a sustainable and long-term safeguarding of CWR.

#### 6.6.1. National Level

There are a number of steps that need to be addressed at the national level to achieve effective, efficient and long-lasting conservation of CWR. The FAO published voluntary guidelines on the conservation of CWRs and wild food plants that provide an overview of all relevant steps that should be considered while planning and implementing conservation activities [19]. Some of these steps are mentioned in the following list:

1. Establishment of a comprehensive picture of the national botanic diversity;
2. Elaborating a national CWR checklist and inventory, e.g., [38,102] and, in parallel, ensuring an adequate integration of CWR conservation with broader national ecosystem, habitat and species conservation plans;
3. Prioritization of CWR taxa/diversity;
4. Eco-geographic and genetic diversity analysis of the priority CWR taxa;
5. Identification of threats to priority CWR taxa and important CWR areas;
6. Gap analysis and establishment of CWR conservation goals;
7. Development of in situ/ex situ CWR national conservation actions [50,103], in accordance with the other forms of conservation, mentioned in point 2 above;
8. Identification of key national CWR protected areas based on gap analysis, on the CWR inventory and occurrence data, the threat status as well as of CWRs under-represented in genebanks;
9. Establishment of national CWR genetic reserves as well as of targeted CWR ex situ collections; and
10. Elaboration of concrete suggestions on how to strengthen utilization, research and education.

A helpful website in preparing and implementing CWR checklists and inventories, as well as conservation strategies, might be the ‘CWR Global Portal’, established and updated by Bioversity International (now called the Alliance of Bioversity International and CIAT) [104]. It provides access to the Interactive toolkit for CWR conservation planning [56]. Guidelines and tools that can support national CWR documentation, prioritization, conservation planning, and implementation are summarized in Supplementary Materials Table S1.

A close collaboration between the national PGRFA program and those concerned with protected areas in a given country will be indispensable to avoid mistakes, to ensure that the best possible management approaches are being used, and that the existing strengths spread over people and institutions are being combined for successful implementation of in situ conservation. This collaboration can also address concerns that typically only a limited number of CWR species is included in protected areas.

#### 6.6.2. Local Level

The national CWR conservation approach will obviously have to address and include the local level actors’ roles and responsibilities. However, often there is very limited published information on specific aspects at the local level that could be included in the planning and implementation processes [55]. A number of obvious aspects can be listed, including the involvement (and active engagement) of all relevant stakeholders in the preparation of management plans for target species. This is a crucial prerequisite when the CWRs are part of a protected area that can no longer be used, for instance, for collecting fresh fruits by the local communities in the neighborhood of such an area. Maxted and Kell [25] included the way to involve local communities in their report as a research question. They also propose an interesting approach in promoting CWR in situ conservation in less formally designated protected areas such as Indigenous and Community Conserved Areas (ICCAs). For the latter, see IUCN [105]. ICCAs are areas where indigenous peoples and local communities have conserved, for millennia, natural environments and species for economic (as well as cultural, spiritual, and aesthetic) reasons, independent of more formal conservation sector interventions. Brooks et al. [106] note that the establishment of genetic or other kinds of reserves for CWRs in areas not yet under protection in times of rapidly rising human population, climate change, and ecosystem instability is a complex goal, which necessitates a carefully researched strategic approach. Sites competing for reserve status would need to be assessed and prioritized for their longer-term sustainability, in terms of the predicted impact of climate change on the site and the economic development plans associated with local communities as well as at the national level [107].

#### 6.6.3. Global Level

Dilemmas with CWRs: Distribution areas of CWR species (at least those of the major food crops) in the tropics/subtropics are, to a large extent, located in countries with limited financial and/or technological resources, limited conservation programs, limited legal frameworks, few breeding program, and which can derive little direct benefits from CWR conservation (especially for local communities). In contrast, interest in these species is largely found in ‘the North’ where financial and technological resources are ample, knowledge is advanced, and where most of the breeding happens. Access to these species, however, is often limited and thus their use in breeding and research for global benefit difficult. Possibly, the only real solution would be to agree within the framework of the existing global instruments, in particular, the FAO Commission on Genetic Resources for Food and Agriculture and the International Treaty, to accord a high(er) priority to the conservation and sustainable use of these threatened resources, to study them more extensively, and to make the diversity freely available as foreseen by these instruments. A mechanism to enable the badly needed global coordination and facilitation of the frequently complex conservation activities, as well as to provide a platform for identifying and prioritizing research activities on CWRs, would be an important help in effectively and efficiently conserving and sustainably utilizing CWRs.

## 7. Conclusions

CWRs have been identified as threatened resources that are understudied, not properly conserved, and that possess a tremendous potential for the breeding of our crops. The latter is particularly important because of climate change, which calls for the urgent development of better adapted crops and varieties for the changing growing conditions in our vulnerable production systems. The protection of the environment is yet another important consideration that can be achieved, or at least important contributions can be made through the increase of crops and varieties that require less harmful inputs and provide still stable and high production levels.

In the above text, we distilled a number of actions that are recommended to be implemented at the various levels, whenever possible, in a timely and collaborative manner. Whereas a number of these recommendations can be implemented by individual countries, others will require agreement and coordination at the global level, where possible, using existing mechanisms and instruments.

### 7.1. Documentation

1. Collating, creating, and sharing more information and knowledge on CWR species, in particular, by stimulating and conducting more research.
2. Establishing national databases and inventories to enable better coordination and implementation of CWR conservation.
3. Developing a global data portal/platform for the exchange and provision of CWR data and information, including tools and guidelines that will facilitate a better coordinated and more efficient conservation, worldwide.

### 7.2. In Situ Conservation

1. Facilitating and encouraging the inclusion of CWRs in national and local conservation agendas and ensuring that they are being given an adequate priority supported possibly by a longer-term financial and organizational structure.
2. Complementing the management and monitoring of CWR in situ conservation sites and genetic reserves with ex situ conservation efforts of the priority species.
3. Identifying existing and novel mechanisms to finance and govern the proposed global coordination and facilitation of CWR in situ conservation should be of high priority. The proposed global network could play an important role in setting standards, sharing experiences, and providing the platform for monitoring and coordination and, thus, to provide a fundamental basis for ensuring our future food security.
4. Increasing the awareness and recognition among actors, especially within the environmental sector, about CWRs as important group of wild species that need to be conserved.

### 7.3. Ex Situ Conservation

1. Ensuring adequate ex situ conservation of threatened national priority CWRs.
2. Ensuring adequate ex situ conservation of a globally agreed list of priority CWRs (e.g., [29]) through national/regional/international genebanks, in particular those that already have global or regional conservation responsibilities for the corresponding crop genepools.
3. The identification and/or application of new methods to assess the viability of seeds, not requiring seed germination tests, could address current difficulties with viability tests and with small seed samples.
4. Large-scale research on CWR seed biology can lead to methods allowing for long-term storage of seeds of these species in genebanks. One such specific research area is the use of cryopreservation for long-term conservation.

### 7.4. Complementary Conservation and Collaboration

1. Development of a generic decision tree on complementary conservation approaches that can be applied to individual CWR species. Supporting guidelines should be developed to facilitate the application of the decision tree and the subsequent implementation of the conservation efforts, using gained experiences with individual species and cases as a basis.
2. Ensuring ready access to the genetic resources and related information, both from in situ as well as ex situ conservation within the framework of existing legal instruments.
3. Facilitating and coordinating phenotypic and molecular characterization of the priority CWRs to provide a basis for pre-breeding and breeding activities through the involvement of conservation, research, and breeding stakeholders.
4. Facilitating/strengthening the collaboration between stakeholders for more effective and efficient conservation, research and use of CWRs as well as to facilitate the transfer of technologies at the local, national, regional, and global levels.

### 7.5. Conservation System

1. Increasing awareness on the importance of and threat to CWRs, including through the active involvement of botanic gardens to ‘demonstrate’ this genetic wealth and the relationship between the CWRs and crop species.
2. Facilitating the training of staff on skills that strengthen the implementation of the above activity areas.
3. Providing a more stable organizational and financial basis for CWR conservation at national level.

## Supplementary Materials

The following are available online at https://www.mdpi.com/2223-7747/9/8/968/s1, Table S1: Guidelines and tools for CWR conservation, including references [108,109,110,111,112].
