# Supplementary material for: Main Challenges and Actions Needed to Improve Conservation and Sustainable Use of Our Crop Wild Relatives
Source: Plants (Basel). 2020 Jul 30;9(8):968. doi: 10.3390/plants9080968 (PMC7463933; doi:10.3390/plants9080968)
Supplement: Supplementary file 1 [file plants-09-00968-s001.pdf]

Table S1: Guidelines and tools for crop wild relative (CWR) conservation

Title[1]	Content	Type	Publisher/ source	Language	Availability
Voluntary Guidelines for the Conservation and Sustainable Use of Crop Wild Relatives and Wild Food Plants[2]	Primarily for use by governments to develop a national plan for the conservation and sustainable use of CWR and wild food plants.	Publication	FAO	EN, FR, ES, AR	Available for download: http://www.fao.org/publications/card/en/c/8f366de9-08a8-42ad-aae1-4f8f6822420e/
CWR checklists, strategies, action plans	List of CWR checklists and strategies	Webpage	Bioversity International	EN	http://www.cropwildrelatives.org/cwr-strategies/
Crop Wild Relatives - A manual of *in situ* conservation[3]	Captures important practical experiences of CWR *in situ* conservation in Armenia, Bolivia, Madagascar, Sri Lanka and Uzbekistan.	Publication	Earthscan	EN, FR, ES	Available for download: http://www.cropwildrelatives.org/resources/in-situ-conservation-manual/
Interactive toolkit for CWR conservation planning[4]	Provides guidance to plan and implement active *in situ* and *ex situ* conservation of CWR at national level.	Website	Bioversity International, University of Birmingham	EN	http://www.cropwildrelatives.org/conservation-toolkit/
Core Descriptors for In Situ Conservation of Crop Wild Relatives, v.1.[5]	The descriptors are designed to facilitate the compilation and exchange of data for CWR *in situ* conservation activities.	Publication	Bioversity International	EN	Available for download: https://www.bioversityinternational.org/e-library/publications/detail/core-descriptors-for-in-situ-conservation-of-crop-wild-relatives-v1/
Crop Wild Relative Checklist and Inventory	Descriptors for the standardized development of CWR checklists and	Publication	Bioversity International	EN	Available for download: https://www.bioversityinternational.org/e-

[1] The footnotes contain the full citation of the respective resource; the numbers in square brackets refer to the reference number in the reference list of the review paper.

[2] FAO *Voluntary Guidelines for the Conservation and Sustainable Use of Crop Wild Relatives and Wild Food Plants*. Food and Agriculture Organization of the United Nations, Rome, Italy, 2017, 106p. [19]

[3] Hunter, D.; Heywood V. (ed.). *Crop Wild Relatives. A Manual of in situ Conservation*. Routledge, London, UK, 2011, 414p. [55]

[4] Magos Brehm J.; Kell S.; Thormann I.; Gaisberger H.; Dulloo E.; Maxted N. Interactive Toolkit for Crop Wild Relative Conservation Planning Version 1.0. University of Birmingham, Birmingham, UK, Bioversity International, Rome, Italy, 2017a. Available at http://www.cropwildrelatives.org/conservation-toolkit/. [56]

[5] Thormann, I.; Alercia, A.; Dulloo, M.E. *Core descriptors for in situ conservation of crop wild relatives v.1.* Bioversity International, Rome, Italy, 2013, 28p. [60]

Descriptors v.1[6]	inventories				library/publications/detail/crop-wild-relative-checklist-and-inventory-descriptors-v1/
Template for the Preparation of a National Strategic Action Plan for the Conservation and Sustainable Use of Crop Wild Relatives[7]	Template to assist countries in preparing their National Strategic Action Plans or National Strategies for the conservation and sustainable use of CWR.	Word file	Bioversity International	EN	Available for download: https://dataverse.harvard.edu/dataset.xhtml?persistentId=doi:10.7910/DVN/QH9XWB
Template for the Preparation of a Technical Background Document for a National Strategic Action Plan for the Conservation and Sustainable Use of Crop Wild Relatives[8]	To assist countries in documenting and detailing the scientific aspects of the development of National Strategic Action Plans or National Strategies for the conservation and sustainable use of CWR.	Word file	Bioversity International	EN	Available for download: https://dataverse.harvard.edu/dataset.xhtml?persistentId=doi:10.7910/DVN/VQVDFA
CWR Checklist and Inventory Data Template v.1[9]	Excel template to help users to establish a CWR checklist and inventory in a systematic manner.	Excel file	Bioversity International	EN	Available for download: https://dataverse.harvard.edu/dataset.xhtml?persistentId=doi:10.7910/DVN/B8YOQL
Occurrence Data Collation Template v.1[10]	Assist users to collate and standardize plant species' occurrence data to perform data analyses needed for conservation planning, including facilitating the formatting of data required for the CAPFITOGEN tools.	Excel file	Bioversity International	EN	Available for download: https://dataverse.harvard.edu/dataset.xhtml?persistentId=doi:10.7910/DVN/5B9IV5

[6] Bioversity International; University of Birmingham. Crop wild relative checklist and inventory descriptors, v.1. Bioversity International, Rome, Italy, 2017, 26p. [64]

[7] Dulloo, M.E.; Magos Brehm, J.; Kell, S.; Thormann, I.; Maxted, N. Template for the Preparation of a National Strategic Action Plan for the Conservation and Sustainable Use of Crop Wild Relatives. https://doi.org/10.7910/DVN/QH9XWB, Harvard Dataverse, V1. 2017, 23p. [108]

[8] Magos Brehm, J.;Kell, S.; Thormann, I.; Maxted, N.; Dulloo, E. Template for the Preparation of a Technical Background Document for a National Strategic Action Plan for the Conservation and Sustainable Use of Crop Wild Relatives, doi: 10.7910/DVN/VQVDFA, Harvard Dataverse, 2017b, 23p. [109]

[9] Thormann, I.; Kell, S.; Magos Brehm, J.; Dulloo, M.E.; Maxted, N. CWR Checklist and Inventory Data Template v.1, doi:10.7910/DVN/B8YOQL, Harvard Dataverse, 2017. [61]

[10] Magos Brehm, J.; Kell, S.; Thormann, I.; Gaisberger, H.; Dulloo, E.; Maxted, N. Occurrence Data Collation Template v.1, doi: 10.7910/DVN/5B9IV5 , Harvard Dataverse, 2017. [62]

Ecogeographic surveys[11]	Synthesis of new knowledge, procedures, best practices and references for collecting plant diversity	Publication (book chapter)	Bioversity International	EN	Available for download: https://cropgenebank.sgrp.cgiar.org/index.php?option=com_content&view=article&id=679
CAPFITOGEN tools[12]	Suite of tools to support the analysis of geographical data and analyses	Manuals and computer application	FAO	EN, ES	http://www.capfitogen.net/
Harlan and de Wet inventory[13]	Priority list of ca. 1,400 CWR species, along with key ancillary data (regional and national occurrence, seed storage behaviour, and herbaria housing major collections of CWR)	Online database	Crop Trust	EN	https://www.cwrdiversity.org/checklist/
Crop Wild Relative Global Occurrence Database[14]	Searchable and downloadable occurrence data on crop wild relatives and crops based on the taxa included in the Harlan and de Wet inventory.	Online database	Crop Trust	EN	https://www.cwrdiversity.org/checklist/cwr-occurrences.php. The entire dataset is downloadable from the GBIF Website at https://www.gbif.org/dataset/07044577-bd82-4089-9f3a-f4a9d2170b2e

[11] Castañeda Álvarez, N.P.; Vincent, H.A.; Kell, S.P.; Eastwood, R.J.; Maxted, N. Ecogeographic surveys. In: Guarino, L, Ramanatha Rao, V, Goldberg, E (eds) Collecting Plant Genetic Diversity: Technical Guidelines. Bioversity International, Rome, Italy 2011 update., 23pp [110]

[12] Parra-Quijano, M.; Torres, E.; Iriondo, J.M.; López, F. CAPFITOGEN Tools User Manual, Version 2.0. Rome: International Treaty on Plant Genetic Resources for Food and Agriculture, Food and Agriculture Organization of the United Nations, Rome, Italy, 2016, 260pp. [111]

[13] Vincent, H.; Wiersema, J.; Dobbie, S.; Kell, S.P.; Fielder, H.; Castañeda-Álvarez, N.P.; P, Eastwood, R.; Guarino, L.; Maxted, N. A prioritized crop wild relative inventory to help underpin global food security. Biol. Cons. 2013, 167, pp 265-275. [29]

[14] Centro Internacional de Agricultura Tropical - CIAT. A global database for the distributions of crop wild relatives. Version 1.12. Occurrence dataset https://doi.org/10.15468/jyrthk accessed via GBIF.org on 2020-06-28, Cali, Colombia, 2018. [112]
